# Heterologous Booster Dose with CORBEVAX following Primary Vaccination with COVISHIELD Enhances Protection against SARS-CoV-2

**DOI:** 10.3390/vaccines10122146

**Published:** 2022-12-14

**Authors:** Shashidhar Jaggaiahgari, Apoorva Munigela, Sasikala Mitnala, Deepika Gujjarlapudi, Venu Simhadri, Nageshwar Reddy D

**Affiliations:** 1 Institute of Translational Research, Asian Healthcare Foundation, AIG Hospitals, Hyderabad 500032, India; 2 Department of Internal Medicine, AIG Hospitals, Hyderabad 500032, India; 3 Department of Biochemistry, AIG Hospitals, Hyderabad 500032, India

**Keywords:** SARS-CoV-2, COVID-19 vaccination, booster dose, Corbevax, Covishield, antibody response, memory cells

## Abstract

Despite effective vaccination programs, waning immunity in the vaccinated populations and the emergence of variants of concern posed a risk of breakthrough infections. A booster dose was demonstrated to provide substantially increased protection against symptomatic disease and hospitalization. We aimed to evaluate immune memory and the efficacy of reducing the rate of SARS-CoV-2 infection post heterologous booster with CORBEVAX after primary vaccination with two doses of COVISHIELD. SARS-CoV-2 S1/S2 spike IgG and RBD-specific antibody responses were elicited with both booster vaccines, with a greater response in individuals receiving heterologous booster. T and B memory responses were increased with booster dose, whereas B memory needed a longer duration to develop in individuals who received a homologous booster (90 days) in comparison to a heterologous booster (30 days). RBD-specific B memory and antibody-secreting (non-memory) B lymphocytes were enhanced with both boosters; however, the duration of response was longer with the heterologous booster compared to the homologous, indicating greater protection with the heterologous booster. The rate of infection 14 days after administration of the heterologous booster was comparatively lower than that of the homologous booster, with the symptoms being much less or asymptomatic.

## 1. Introduction

COVID-19 vaccination programs have remarkably reduced SARS-CoV-2 infections and the severity of the disease [1,2]. The vaccines against SARS-CoV-2 that are widely administered are the adenoviral vector-based Oxford-AstraZeneca (ChAdOx1-nCoV-19; Covishield in India), the mRNA vaccines BNT162b (BNT-Pfizer), and mRNA-1273 (Moderna), apart from others. India produced two indigenously developed vaccines; Covaxin by Bharat Biotech (BBV 152) in association with the Indian Council of Medical Research and Corbevax (BECOV2D) by Biological E. Limited (Bio.E) in collaboration with the Baylor College of Medicine in Houston, United States, and American company Dynavax Technologies (DVAX). Despite effective vaccination programs, waning immunity in the vaccinated populations [3,4] and the emergence of variants of concern posed a risk of breakthrough infections [5]. Frontline workers, individuals with comorbidities, and those above 50 years of age are at a greater risk of breakthrough infections. As early as September 2021, a few European countries had witnessed a surge in SARS-CoV-2 infections with associated fatalities in fully vaccinated individuals [6]. Booster doses were believed to enhance protection against severe COVID-19 and were approved by various countries for administration. The UK, Germany, France, and Israel were the first countries to administer booster doses to their populations [7,8]. A booster dose was demonstrated to provide substantially increased protection against symptomatic disease and hospitalization in those aged ≥50 years [9,10]. At the time of approval for booster dose administration, only a few developed countries completed vaccinating their entire population. Therefore, it was opined that there would be a humongous need for vaccine doses that needed scaling up of production.

Although massive efforts were made to manufacture vaccines, shortage of doses and distribution disparity enforced several challenges in vaccinating the world’s population, increasing the need for adapting vaccine combination regimens [11]. Research has dependably reported a combination of vaccines to provide similar strong antibody response/T lymphocyte responses as homologous vaccines and higher SARS-CoV-2-specific CD-8 T lymphocyte levels [12]. Antibodies elicited after heterologous vaccines were shown to exhibit neutralizing activity against the SARS-CoV-2 wild-type virus as well as variants of concern (VOC) [13,14]. In addition, previously, we have demonstrated a higher neutralizing antibody response and protective immunity with a single dose of vaccination in previously infected individuals [15]. We had conducted a pilot study to assess the safety and immunogenicity of a combination of vaccines with Covishield and Covaxin in the primary vaccination regimen. It was observed that a combination of vaccines elicited greater immune responses [16]. A higher immunogenicity was observed in individuals who received a heterologous booster compared to a homologous booster, with similar reactogenicity and adverse events. [17]. Subsequent to several reports of waning immunity, a homologous booster dose was approved by the Indian Government in December 2021. Corbevax is the first vaccine authorized to be given as a heterologous booster dose in June 2022. Covishield is a recombinant, replication-deficient chimpanzee adenovirus vector that encodes the entire S protein (SARS-CoV-2 Spike (S) glycoprotein), which is produced in the genetically modified human embryonic kidney (HEK) 293 cells, whereas Corbevax is a protein-based vaccine that contains protein subunit of receptor-binding domain (RBD) from the spike protein of SARS-CoV-2, which is produced in the yeast *Pichia pastoris*. Since efficacy data on the heterologous booster vaccine are limited from India, we intended to assess the immunogenicity and immune memory of the Corbevax heterologous booster dose in healthcare workers who had received Covishield as a primary vaccine regimen by comparing it with the homologous Covishield booster dose. The objective of the study was to evaluate the immunogenicity/immune memory of a single booster dose of the Corbevax vaccine when administered to healthcare workers previously vaccinated with two doses of Covishield at least 6 months prior and its efficacy in terms of reducing the rate of infection.

## 2. Materials and Methods

### 2.1. Study Participants

This study protocol was approved by Institutional Ethical Committee of AIG Hospitals and was registered with clinical trials registry of India (CTRI/2022/02/040563). Participants aged between 18 and 70 years with or without prior SARS-CoV-2 infection and have completed 6 months of second dose of primary vaccination were included. Participants with chronic illness, on immunosuppressive drugs, pregnant women, and those who have not completed 6 months of primary vaccination were excluded. All the participants had given written informed consent. Whole Blood (3 mL) was collected at baseline prior to administering booster dose (pre), one month (day 30), and three months (day 90) after booster dose to assess S1/S2 spike IgG antibody and immune memory T and B lymphocyte responses. The participants were followed for up to 28 days to assess the reactogenicity and adverse events if any such as thrombocytopenia, ischemic cardiac and cerebrovascular events after a booster dose with Corbevax following 2 doses of Covishield as primary vaccination. The participants were also followed for 6 months for SARS-CoV-2 infections and symptomatic disease.

### 2.2. Enumeration of SARS-CoV-2 S1/S2 Spike IgG Antibody Titre

SARS-CoV-2 S1/S2 spike IgG antibodies were enumerated employing LIAISON SARS-CoV-2 S1/S2 IgG assay, a chemiluminescence immunoassay (CLIA) for quantitative detection of anti-S1 and anti-S2 IgG antibodies of SARS-CoV-2 following manufacture instructions (LIAISON^®^ XL, DiaSorin, Saluggia, Italy). Concentrations of <12.0 AU/mL are interpreted as negative, ≥12.0 to <15.0 AU/mL are interpreted as equivocal, and ≥15.0 AU/mL are interpreted as positive [18].

### 2.3. Evaluation of RBD-Specific Antibody Titer

RBD-specific antibody titers were evaluated using Elecsys Anti-SARS-CoV-2 S assay. The Elecsys Anti-SARS-CoV-2 S immunoassay is an Electrochemiluminescence immunoassay (ECLIA) intended for quantitative estimation that detects high-affinity antibodies to the SARS-CoV-2 S protein RBD and measured on cobas^®^ e601 modular analyzers (Roche Diagnostics, Rotkreuz, Switzerland). Results were reported as the analyte concentration of each sample in U/mL, with <0.80 U/mL interpreted as negative for anti-SARS-CoV-2 S antibodies and ≥0.80 U/mL interpreted as positive for anti-SARS-CoV-2 S antibodies [19].

### 2.4. Estimation of Immunological T and B Memory Cells

Peripheral blood mononuclear cells (PBMCs) isolated were characterized phenotypically by flow cytometry for T and B Lymphocytes along with COVID-19 RBD-specific B memory and non-memory B Lymphocytes. T lymphocytes were characterized using CD3 (APC-H7), CD4 (Percp Cy 5.5), CD8 (FITC), CD45RA (PE-Cy 7), CCR7 (BV421), and B lymphocytes were enumerated by CD20(V500), CD27 (PE). RBD-specific B lymphocytes were enumerated using Biotinylated RBD conjugated with APC Streptavidin from BD Biosciences. Briefly, PBMCs were stained with T and B lymphocyte antibody staining mix and incubated at RT for 30 min. Prior to adding antibody mix, Biotinylated RBD was conjugated with APC Streptavidin for 30 min at 4–8 °C. After Incubation cells were washed with staining buffer and data were acquired on FACS Lyrics flow cytometer (BD BIOSCIENCES) Appropriate isotype-matched, non-reactive fluorochrome-conjugated antibodies were employed as controls [20].

### 2.5. Statistical Analysis

A database was generated in MS Excel and all analyses were carried out using the Statistical Package for Social Sciences (SPSS Version 20, IBM, Chicago, IL, USA) software. Continuous variables were expressed as mean (95% confidence interval; CI) and categorical variables as proportions. Variables were compared using Student’s *t*-test and one-way Annova. A two-tailed ‘*p*’ value of ≤0.05 was considered statistically significant.

## 3. Results

### 3.1. Participant Outcomes

The study included a total of 250 healthcare workers from the Asian Institute of Gastroenterology Hospitals in Hyderabad, India. Of these, 63.2% (158/250; mean age: 38.8 ± 11.8 years) were males, and 36.8% (92/250; mean age: 33.6 ± 10.1 years) were females. Among them, 39 (15.6%) were previously infected with SARS-CoV-2. All 250 participants had received 2 doses of Covishield as the primary vaccine regimen.

### 3.2. Heterologous Corbevax Booster Dose after Covishield Priming Did Not Evoke Reactogenicity and Adverse Events

Single-dose Corbevax administration did not evoke any adverse events in any of the participants who had received Covishield as their primary vaccination assessed up to day 28 post-booster as with homologous booster Covishield in our study population. Mild reactogenicity was seen only in previously infected individuals who experienced headaches lasting for a few hours. Pain at the injection site was experienced by all the participants, irrespective of the booster dose.

### 3.3. Heterologous and Homologous Booster Doses Elicited Significant Immune Responses

**Figure 1:**
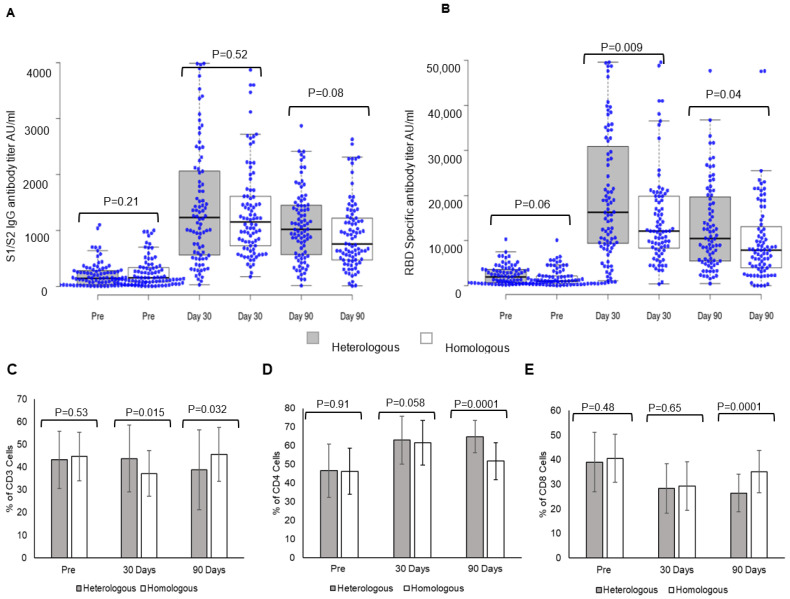

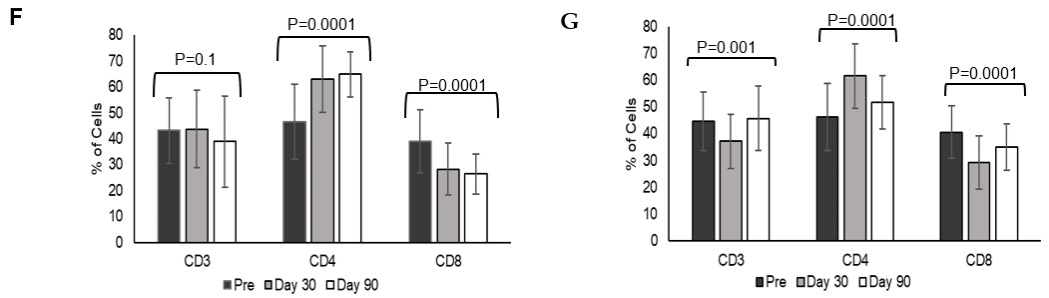
Booster dose vaccine elicited antibody response and immune cell response: Antibody and immune cells response in individuals receiving heterologous and homologous booster vaccine. (**A**) Comparative evaluation of SARS-CoV-2 S1/S2 spike IgG antibodies before (pre) and after booster vaccination at day 30 and day 90 with Corbevax (Heterologous) and Covishield (Homologous). (**B**) SARS-CoV-2 RBD-specific antibodies comparative evaluation before (pre) and after booster vaccination day 30 and day 90 with Corbevax (Heterologous) and Covishield (Homologous). Comparison of (**C**) CD3, (**D**) CD4, and (**E**) CD8 in between the hetero and homologous groups at pre, day 30, and day 90 and *p* values were compared in between the groups (**A**–**E**). Immune cell responses in individuals receiving booster doses—(**F**) CD3, CD4, CD8 T Lymphocytes in individuals receiving heterologous booster dose with Corbevax (**G**) CD3, CD4, CD8 T Lymphocytes in individuals receiving homologous booster dose with Covishield and *p* values were shown together for pre, Day 30 and day 90 (**F**,**G**).

Heterologous and homologous boosters elicited a strong S1/S2 spike IgG antibody response (Figure 1). Post-booster levels at day 30 were 1413 ± 1080 AU/mL vs. pre-booster levels of 207.8 ± 227.11 AU/mL in individuals receiving heterologous boosters, while in those receiving homologous boosters (Figure 1A), antibody levels were 1331.6 ± 832.5 AU/mL vs. pre-booster levels of 265.95 ± 268.39 AU/mL. The comparison of S1/S2 spike IgG antibody responses between the two groups showed no significant difference at day 30 (*p* = 0.52). The antibody levels declined at day 90 in both booster dose groups; heterologous 1012.74 ± 672.02 AU/mL, homologous 978.37 ± 618.2 AU/mL, and the decline was greater in individuals receiving homologous booster. S1/S2-spike protein IgG antibody responses within the group are given in the supplementary section (Figure S1).

A comparison of RBD-specific antibody response between heterologous and homologous booster doses showed a significantly higher response in individuals receiving heterologous booster with Corbevax after two doses of Covishield (Figure 1B). Post-booster levels at day 30 in individuals receiving heterologous booster were 19,945 ± 13,605 AU/mL vs. pre-booster levels 2393 ± 2073 AU/mL, while in those receiving homologous booster (Figure 1B), the levels were 15,078 ± 10,633 AU/mL vs. pre-booster levels of 1810 ± 1959 AU/mL. A comparison of RBD-specific antibody titers between individuals receiving heterologous and homologous boosters revealed significantly higher responses in individuals receiving a heterologous booster at day 30 (*p* = 0.009) and day 90 (*p* = 0.04). The antibody levels declined significantly at day 90 in both booster dose groups; heterologous 13,112 ± 9624 AU/mL and homologous 10,172 ± 9167 AU/mL (Figure S1).

### 3.4. Heterologous Booster Dose Elicits Higher CD4 T Lymphocyte Response

A heterologous booster dose with Corbevax after primary vaccination with Covishield evoked immune cell responses as with a homologous booster dose. A significant difference was observed in CD3 T-lymphocyte response at day 30 in individuals receiving a heterologous booster (*p* = 0.015), while the difference was higher in homologous recipients at day 90 (*p* = 0.032) (Figure 1C). CD4-T lymphocyte responses were higher at day 30 and day 90 in individuals receiving heterologous booster with Corbevax in comparison to individuals receiving homologous booster with Covishield (Figure 1D). While CD4-T lymphocyte increased at day 30, (62.92 ± 12.8) and remained consistent until day 90 (64.85 ± 8.72) (*p* = 0.0001) in individuals who received heterologous booster with Corbevax, CD4-T lymphocyte response increased at day 30 (day 30, 66.2 ± 12.4) as compared to pre-booster dose (day 0, 46.2 ± 12.43) (Figure 1F) and decreased in individuals who received homologous booster Covishield at day 90 (day 90 51.66 ± 10.03) (*p* = 0.0001) (Figure 1G). CD8 T-lymphocyte responses decreased in all the individuals, irrespective of whether they received a homologous or a heterologous booster dose at day 90 (Figure 1E–G).

### 3.5. Differential Immune Memory Responses after Heterologous and Homologous Booster Doses

**Figure 2:**
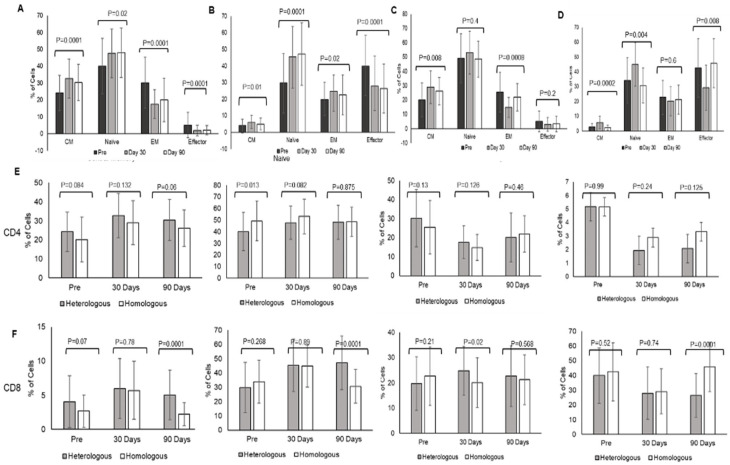
Immunological memory: Corbevax vs. Covishield in T lymphocytes: T memory immune response analysis of (**A**) CD4 and (**B**) CD8 in heterologous booster, (**C**) CD4 (**D**) CD8 in homologous booster employing flow cytometry for central memory (CM) CCR+CD45RA−, Naïve CCR7+CD45RA+, effector memory (EM) CCR7−CD45RA− effector (Eff) CCR7−CD45RA+ populations and *p* values were shown together for pre, day 30, and day 90. Differential immune memory responses comparison of heterologous and homologous booster doses together in (**E**) CD4 and (**F**) CD8 T lymphocytes and *p* values were compared in between the groups.

Heterologous booster dose elicited an increase in CD4 = T lymphocytes Central memory at day 30 (CM 32.79 ± 11.6, Naïve 47.6 ± 14.3) and day 90 (CM 30.44 ± 10.7, Naïve 48.06 ± 14.6) in comparison to pre-booster levels (CM 24.24 ± 10.4, Naïve 40.14 ± 10.4) (CM *p* = 0.0001, Naïve *p* = 0.002). Effector memory (EM) and effector cells (Eff) decreased at day 30 (EM 17.6 ± 8.5, Eff 1.95 ± 3.15), and day 90 (EM 20.09 ± 12.84, Eff 2.08 ± 2.76) post-booster in comparison to pre-booster levels (EM 30.3 ± 15.08, Eff 5.17 ± 7.54) (EM *p* = 0.0001, Eff *p* = 0.0001). (Figure 2A).

Homologous booster dose with Covishield evoked a significant increase in CD4 + T lymphocyte central memory (CM) at day 30 (28.97 ± 11.72) and at day 90 (26.15 ± 9.5) in comparison to pre-booster levels (20.12 ± 11.7) (*p* = 0.008). Naïve CD4 T-lymphocytes decreased at day 90 (48.54 ± 12.6) in comparison to day 30 (53.17 ± 14.9) (*p* = 0.4). Effector memory (EM) and effector cells (Eff) decreased at day 30 (EM 14.89 ± 6.9, Eff 2.98 ± 4.8) and day 90 (EM 21.98 ± 9.6, Eff 3.3 ± 5.3) post-booster in comparison to pre-booster levels (EM 25.49 ± 13.9, Eff 5.16 ± 7.7.17) (EM *p* = 0.0008, Eff *p* < 0.2) (Figure 2B).

Comparing the two boosters indicated that both boosters induced CD4+ T lymphocyte central memory (CM) that increased at day 30 (heterologous—pre-booster 24.24 ± 10.4; post-booster day 30–32.79 ± 11.6) and day 90 (30.44 ± 10.7); (homologous pre-booster 20.12 ± 11.7, day 30—28.97 ± 11.51, day 90—26.15 ± 10.69). The difference was not significant between heterologous and homologous groups. There was no significant change in naïve CD4 + T lymphocytes, effector memory, and effector lymphocytes at day 30 and day 90 between the groups. Effector memory and effector CD4 + T lymphocytes were decreased at day 30 and day 90 when compared with pre-booster levels in both groups (Figure 2E).

CD8+ T lymphocyte naïve and effector memory cell population increased significantly at day 30 (naïve 45.41 ± 18.34, EM 24.75 ± 9.68) and day 90 (naïve 47.2 ± 18.88, EM 22.62 ± 12.02) post-booster in comparison to pre-booster levels (naïve 29.86 ± 17.56, EM 19.67 ± 10.06) (naïve *p* = 0.0001, EM *p* = 0.02) (Figure 2C). In homologous group analysis CD8+ naïve T lymphocytes increased significantly at day 30 (45.00 ± 14.75) and decreased at day 90 (30.7 ± 11.8) (*p* = 0.004), and there is not much variation in effector memory at day 0, day 30 and 90 (22.64 ± 11.56, 20.08 ± 9.8, 21.2 ± 9.8) (*p* = 0.6) and CM (2.68 ± 2.39, 5.7 ± 4.2, 2.24 ± 1.7) (*p* = 0.0002). whereas effectors were decreased at day 30 (29.1 ± 15.3) in comparison to day 0 (42.5 ± 19.76) and day 90 (45.53 ± 16.6) (*p* = 0.008) (Figure 2D). The comparison of boosters indicated induction of CD8T lymphocyte memory responses with a significant increase in central memory and naïve lymphocytes at day 90 (*p* = 0.0001) in the heterologous group compared to the homologous group (Figure 2F). CD8 effector memory lymphocytes were higher at day 30. The gating strategies for central memory (CCR+CD45RA−), naïve (CCR7+CD45RA+), effector memory (CCR7−CD45RA−), and effector cells (CCR7−CD45RA+) of CD4 and CD8 T lymphocytes are shown in Figure S2.

### 3.6. Heterologous Booster Dose Elicited Total B Lymphocyte Immune Responses

**Figure 3:**
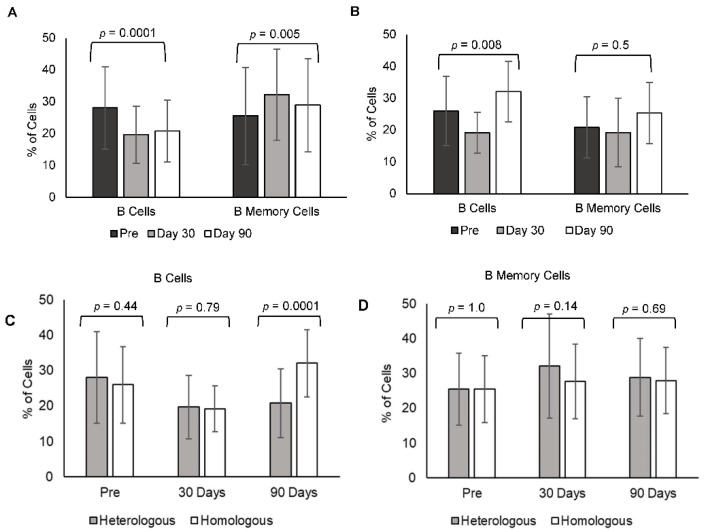
B memory cells heterologous vs. homologous booster dose: humoral immune response in individuals receiving heterologous and homologous booster vaccines. Comparison of (**A**) B lymphocytes (CD20+) and (**B**) B memory (CD20+CD27+) cells at pre, day 30, and day 90 samples of individuals receiving heterologous and homologous booster vaccine *p* values were compared in between the groups. Within group analysis of B cells and B memory cells in (**C**) heterologous and (**D**) homologous booster doses *p* values were shown together for pre, day 30 and day 90.

Heterologous booster induced a decrease in total B lymphocytes (CD20+) at day 30 (19.6 ± 8.9) and day 90 post-booster (20.77 ± 9.73) in comparison to pre-booster levels (28.02 ± 12.9) (*p* = 0.0001). Interestingly, B memory cells (CD27+) increased in post-booster samples at day 30 (32.18 ± 14.37) and day 90 (28.8 ± 14.67) in comparison to pre-booster levels (25.3 ± 15.19) (*p* = 0.005) (Figure 3A). Whereas the homologous booster-induced decrease in total B lymphocytes (CD20+) decreased at day 30 (19.18 ± 6.4) and increased at day 90 (32.06 ± 9.4) in comparison to pre-booster levels (25.02 ± 10.9) (*p* = 0.008). Interestingly, B memory cell (CD27+) levels were not changed in post-booster samples at day 30 (19.23 ± 10.7) but increased at day 90 (25.4 ± 9.5) in comparison to pre-booster levels (20.8 ± 9.63) (*p* = 0.5) (Figure 3B). A comparison of B cells and B memory cells in both heterologous and homologous booster doses together is shown in Figure 3C (B cells) and Figure 3D (B memory cells).

### 3.7. Heterologous Booster Dose Elicits RBD-Specific B Non-Memory (Antibody-Secreting) and B Memory Cells

**Figure 4:**
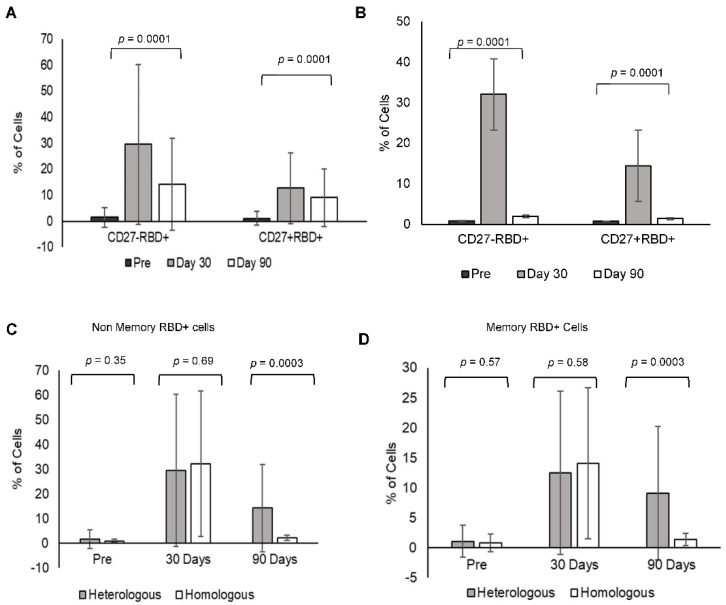
RBD-specific B memory and non-memory: individual analysis of RBD-specific non-memory and RBD-specific memory in heterologous and homologous booster groups at pre, day 30, and day 90 together and *p* values were shown between the groups (**A**,**B**). Comparison of RBD-specific B non-memory (RBD+CD27−) and memory (RBD+CD27+) B lymphocytes in individuals receiving heterologous (**C**) and homologous (**D**) booster vaccine at pre, day 30, and day 90 and *p* values were shown for the same.

Heterologous booster increased RBD-specific B memory cells (CD27+) and B non-memory cells (antibody-secreting B lymphocytes CD27−) at day 30 (RBD+CD27+: 12.67 ± 13.55, RBD+CD27: 29.5 ± 30.8) and day 90 in comparison to pre-booster levels (RBD+CD27+: 1.07 ± 2.65, RBD+CD27−: 1.52 ± 3.8). However, the levels decreased at day 90 in comparison to day 30 (RBD+CD27+: 9.02 ± 11.01, RBD+CD27−: 14.17 ± 17.74) (Figure 4A).

Homologous booster dose increased the RBD-specific B memory cells (CD27+) and B non-memory cells (CD27−) at day 30 (RBD+CD27+: 14.44 ± 12.48, RBD+CD27−: 32.06 ± 29.5) in comparison to pre-booster levels (RBD+CD27+: 0.78 ± 1.4, RBD+CD27−: 0.87 ± 0.63). However, the levels were minimal at day 90 in comparison to day 30 (RBD+CD27+: 1.4 ± 1.04, RBD+CD27−: 2.02 ± 1.08) (Figure 4B). A comparison of RBD-specific memory and non-memory B cells in between the (C) heterologous and (D) homologous groups gating strategy for the same is shown in Figure S3.

### 3.8. CCR7+ and CD27+ Levels in B Lymphocytes and RBD-Specific B Lymphocytes

B lymphocytes were further characterized using CCR7 and CD27 for their expression levels before and after booster doses to confirm the lineage towards antibody-secreting and memory cells. CCR7 plays a major role in directing B lymphocytes to secrete antibodies. CCR7+ cells increased both memory and non-memory B lymphocytes after the booster vaccine. RBD+CD27−CCR7+ non-memory B lymphocytes increased on day 30 after the booster dose (heterologous 86.05 ± 9.01, homologous 83.49 ± 9.83) in comparison to pre-booster levels (heterologous 74.34 ± 14.34, homologous 79.81 ± 11.85), which were maintained up to 90 days (the heterologous booster, 82.43 ± 11.91 and the homologous booster 79.24 ± 10.78). Whereas RBD+CD27+CCR7+ memory B lymphocytes increased at day 30 and were maintained up to 90 days with a heterologous booster (day 30: 73.87 ± 9.19, day 90: 72.05 ± 11.34) compared to pre-booster (66.61 ± 15.81) levels, while in homologous RBD-specific memory B lymphocytes decreased at day 90 (pre-booster 72.68 ± 14.85, day 30 after booster 71.79 ± 10.9, day 90: 66.61 ± 10.7).

### 3.9. Heterologous Booster with Corbevax after Priming with Covishield Reduced SARS-CoV-2 Infection Rate and Symptoms

**Figure 5:**
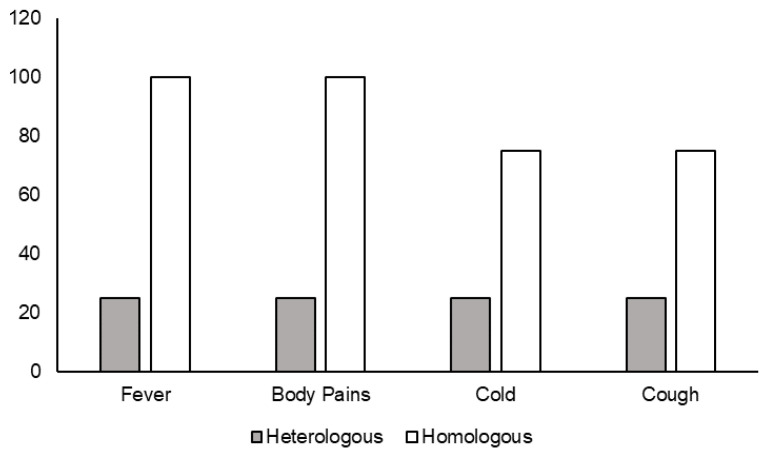
Clinical symptoms in individuals infected 14 days after receiving heterologous and homologous booster vaccines.

Out of 250 individuals who received booster doses, 48 individuals were infected after the booster dose, as follows: only 10 individuals were positive for SARS-CoV-2 after 14 days of receiving the boosters. Among them, four individuals received the heterologous booster Corbevax and six received the homologous booster Covishield. While all six who received a homologous booster developed symptoms such as fever/body pains, a cold, and a cough, only one out of four receiving the heterologous booster with Corbevax developed mild symptoms after the booster dose (Figure 5).

## 4. Discussion

Vaccines were authorized to be given as booster doses to enhance immune protection on 25 December 2021, by the Government of India. Homologous boosters were only approved and were initiated in January 2022 to be given to frontline workers and individuals aged above 60 years who had completed 9 months of primary vaccination. We studied post-booster infection rates in individuals who received Corbevax as a booster and Covishield as a primary vaccine. Our study provides evidence for enhanced immunological T and B lymphocyte memory and reduced infections in individuals who received the heterologous booster Corbevax after priming with 2 doses of Covishield.

Covishield is an Oxford-AstraZenica vaccine manufactured in India by Serum Institute, was the first vaccine to be administered to frontline workers in January 2020. COVAXIN and Corbevax are the two indigenously developed vaccines in India. Corbevax is the first Indian vaccine to be approved as a heterologous booster in June 2022 for administration to the Indian population [21]. While all three vaccines were shown to evoke immune responses, we in a pilot study Ref. [16] and others from different parts of the world have reported combinations of vaccines to induce greater immunogenicity [22,23,24]. Our study corroborates with other studies showing durable humoral and cellular immune responses in individuals who were administered heterologous booster (Ad26.COV2.S) after two doses of mRNA vaccine (BNT162b2) compared to homologous booster vaccine [22,25].

In this study, we show that both homologous (Covishield priming Covishield boosting) and heterologous boosters (Covishield priming Corbevax boosting) induce strong SARS-CoV-2 S1/S2 spike IgG antibody responses. However, our data shows a greater number of individuals had higher antibody titers in individuals receiving heterologous booster in comparison to a homologous booster. The RBD-specific antibody response was much greater with Corbevax administered as a heterologous booster after two doses of Covishield, probably due to the protein nature of the vaccine. These results indicate that more number of people have higher protection with heterologous booster vaccine. Stronger immunogenicity with heterologous boosters could be due to exposure of the immune system to different kinds of antigens, resulting in varied antibody responses that could neutralize the virus more efficiently.

Additionally, we also demonstrate increased CD4 T lymphocyte responses sustaining up to 90 days and beyond in individuals receiving Corbevax as a heterologous booster. Whereas CD4 T lymphocyte responses declined at 90 days in individuals receiving homologous boosters with Covishield. CD4 T lymphocytes are known to have a role in activating B lymphocytes to secrete antibodies and cytotoxic T lymphocytes to secrete effector molecules [26]. The longer duration of CD4 T lymphocyte responses with Corbevax indicates protection for a longer period of time with the heterologous booster dose as compared to the homologous booster. Upon exposure to the virus, CD4 T lymphocytes activate B lymphocytes and CD8 T lymphocytes and provide protection against infection.

The efficacy and effectiveness of vaccines depend upon the strength and duration of vaccine-induced immunogenicity and immune memory. We here show that a heterologous booster with Corbevax induces naïve and central memory cells in CD4T lymphocytes and naïve and effector memory in CD 8 T lymphocytes, which lasted up to day 90. On the contrary, a homologous booster with Covishield induced a significant increase in central memory CD4 T lymphocytes but a decline in naïve CD4 T lymphocytes at day 90. Naïve CD8 T lymphocytes increased at day 30 and declined at day 90, with no significant change in effector or effector memory CD8 T lymphocytes. Antigen/vaccine-induced naïve T lymphocytes develop into memory or effector cells, and the proportion of naïve T lymphocytes determines the strength of the recall response and immune protection upon exposure to the antigen [27]. Our data supports that a heterologous booster with Corbevax enhances naïve CD4/CD8 T lymphocytes and CD8 T lymphocyte effector memory responses, inducing stronger effector immune memory for a longer duration, protecting against infection. Although previous studies have shown the safety and immunogenicity of Corbevax, this study shows enhanced antigen-specific CD4/CD8 naïve and effector memory responses for a longer duration when given as a heterologous booster after priming with Covishield.

Both homologous and heterologous boosters enhanced the total B lymphocyte responses, but B memory needed a longer duration to develop in individuals who received a homologous booster (90 days) in comparison to a heterologous booster (30 days). RBD-specific B memory and antibody-secreting (non-memory) B lymphocytes enhanced with both boosters, which was confirmed by CCR7 expression, however, the duration of response was higher in those receiving heterologous booster. CCR7 expression on B cells allows for effective T and B cell interaction, eliciting a cellular immune response, and it is strongly upregulated in the maturation stages of the B cells [28,29].

The strength of this study is that we followed all these individuals receiving homologous or heterologous boosters for six months. All the participants tolerated the combination of vaccines without any adverse events followed for 28 days indicating priming with Covishield and boosting with Corbevax is safe without causing any adverse events measured. The rate of infection 14 days after administration of the heterologous booster was comparatively lower than that of the homologous booster, even those who developed infection did not develop a symptomatic disease, indicating superior protection against SARS-CoV-2 infection with the heterologous booster.

The limitation of the study is that the convenience sampling method does not use randomization. This was because the Indian Government approved only homologous boosters for all the individuals at the time the study was initiated. The number of people consenting to heterologous was relatively less. Another limitation of the study was that the T-cell responses enumerated were total T-cell responses and not antigen-specific responses. However, the strength of the study was assessing the memory responses and follow-up for infection rates.

In conclusion, we report safety, enhanced protection, and a reduced infection rate with a heterologous booster dose of Corbevax after priming with Covishield.

## Data Availability

The data presented in this study are available on request from the corresponding author.

## Supplementary Materials

The following are available online at https://www.mdpi.com/article/10.3390/vaccines10122146/s1, Figure S1: Booster dose vaccine elicited antibody response and immune cell response: Comparative evaluation of SARS-CoV-2 S1/S2 Spike IgG antibodies before (pre) and after booster vaccination at day 30 and day 90 with (A) Corbevax (Heterologous) and (B) Covishield (Homologous). SARS-CoV-2 RBD specific antibodies comparative evaluation before (pre) and after booster vaccination day 30 and day 90 with (C) Corbevax (Heterologous) and (D) Covishield (Homologous). Figure S2: Gating strategy of CD4 and CD8 memory in both heterologous and homologous booster samples for Central Memory (CM), Naïve, Effector Memory (EM) and Effector (Eff). Figure S3: Gating strategy of RBD specific memory and non-memory B lymphocytes.
