# Supplementary material for: Heterologous Booster Dose with CORBEVAX following Primary Vaccination with COVISHIELD Enhances Protection against SARS-CoV-2
Source: Vaccines (Basel). 2022 Dec 14;10(12):2146. doi: 10.3390/vaccines10122146 (PMC9781398; doi:10.3390/vaccines10122146)
Supplement: Supplementary file 1 [file vaccines-10-02146-s001.zip › vaccines-1931398-supplementary.pdf]

Supplementary Data

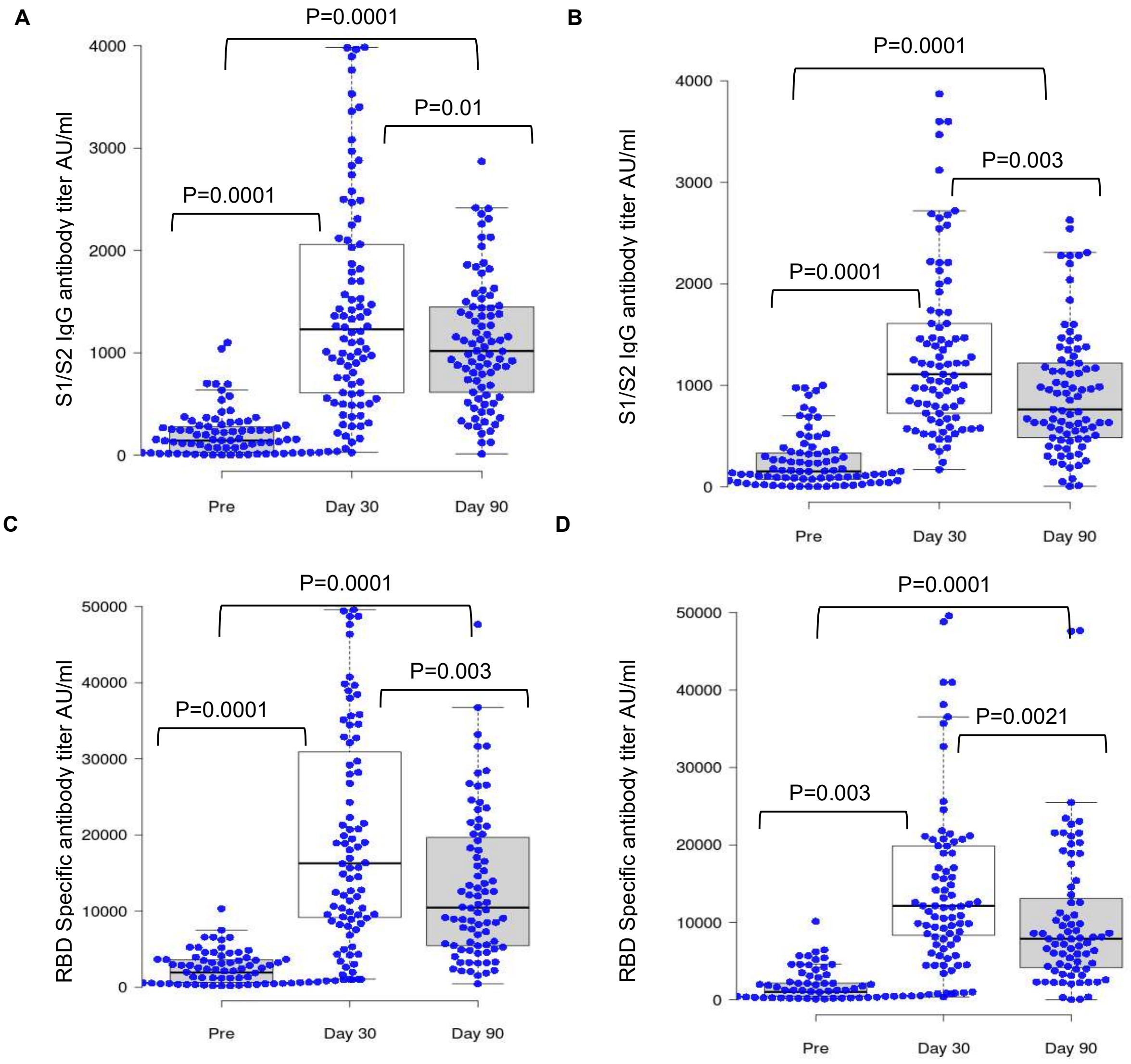

Figure S1. Booster dose vaccine elicited antibody response and immune cell response: Comparative evaluation of SARS-CoV-2 S1/S2 Spike IgG antibodies before (pre) and after booster vaccination at day 30 and day 90 with (A) Corbevax (Heterologous) and (B) Covishield (Homologous). SARS-CoV-2 RBD specific antibodies comparative evaluation before (pre) and after booster vaccination day 30 and day 90 with (C) Corbevax (Heterologous) and (D) Covishield (Homologous).

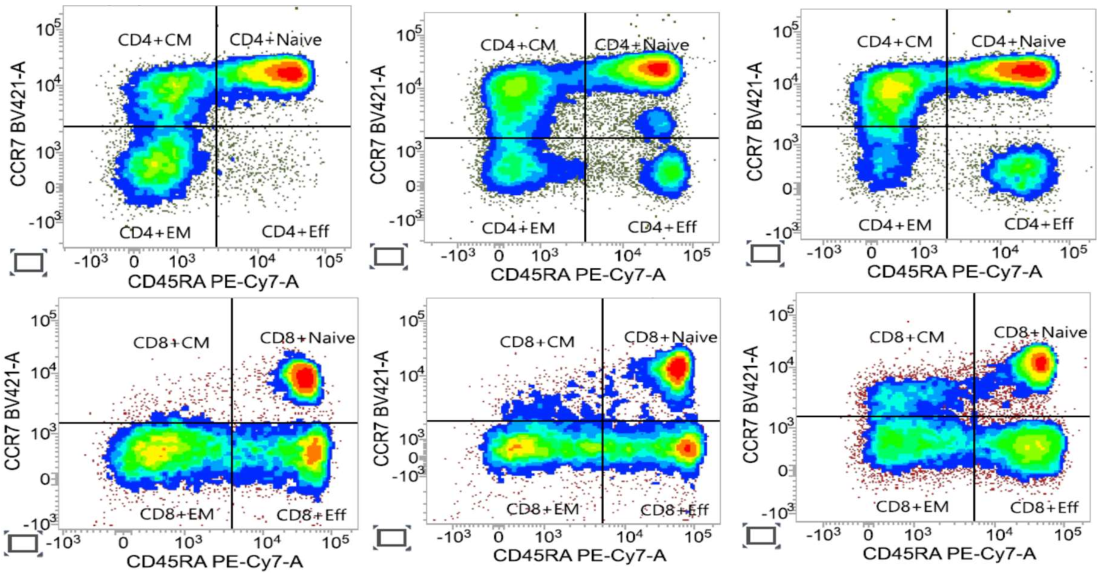

Figure S2. Gating strategy of CD4 and CD8 memory in both heterologous and homologous booster samples for Central Memory (CM), Naïve, Effector Memory (EM) and Effector (Eff).

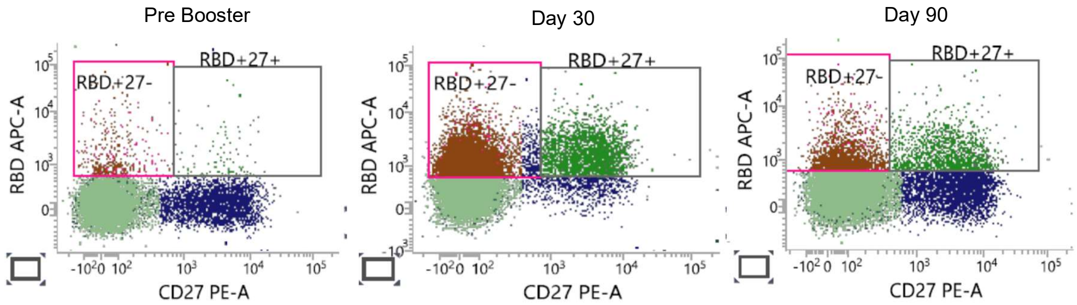

Figure S3. Gating strategy of RBD specific memory and non-memory B lymphocytes.
